# Tobacco advertising and the art and science of persuasion

**DOI:** 10.1186/1617-9625-1-2-95

**Published:** 2002-06-15

## 

"I know a maiden fair to see,

Take care!

She can both false and friendly be,

Beware! Beware!

Trust her not,

She is fooling thee."

Henry Wadsworth Longfellow *Hyperion*

There has been a great deal of tobacco control literature over the past decade regarding how advertising is used to persuade consumers to purchase tobacco, and ultimately to become loyal brand smokers. In a similar manner the literature increasingly describes methods to "counter advertise;" i.e., to use advertising in an attempt to persuade individuals to quit smoking, or better yet, to not initiate smoking. The article by DiFranza and Pollay in this issue of journal raises an intriguing question: What really is advertising and what is its aim? *Advertising*, the noun, is defined in the *American Heritage Dictionary *as "the action of attracting public attention to a product or business." "To make public announcement of; especially, to proclaim the qualities or advantages of (a product or business) so as to increase sales," is the entry for the verb, *advertise*. Derived from the old French *advertir *or in English *advert*, our now modern word *advertising *has its roots in a word meaning, "to turn toward."

As I look at the word and the concept of advertising in light of its definition, I probe a bit further into its underlying theory and research to better understand the nature of advertising in general and tobacco advertising specifically. O'Keefe writes in Persuasion: Theory and Research* that "the advertiser's task is to get the consumer to believe that the product has various attributes thought desirable by the consumer (and, correlatively, to avoid having the consumer believe that the product has seriously undesirable attributes)." This is particularly relevant to tobacco advertising, as advertisers and tobacco executives attempt to portray as desirable a product with many undesirable attributes; e.g., yellow teeth, stained fingers, tobacco smell, face wrinkles, loss of breath, loss of taste, sickness, and death. To those of us in public and community health, these facts are among the most compelling reasons not to smoke or purchase tobacco products, but it creates an incredible challenge to advertisers as they attempt to influence consumers to "turn toward" the product by making its attributes desirable. As a result there have been advertisements that depict various types of "pleasure" associated with smoking. One can think of the "Kools" tobacco advertisements with their play on the word "cool" to convey various desirable attributes such as "moderately cold; neither hot or cold," "to calm down, slow down or relax," or the slang connotation of "composure."

Consequently, tobacco advertising aims to have the consumer believe that the tobacco products have these desirable characteristics while ignoring the very obvious undesirable attributes. This manipulation can especially prey on youth who are psychologically receptive to such messages. Unfortunately, without any counter-advertising efforts by health or regulatory agencies about the dangers of smoking, youth may not have pre-existing beliefs regarding the negative or undesirable aspects of tobacco. Fortunately, in a number of countries such counter-advertising messages are fairly widespread through various media outlets, placing tobacco advertisers in the position of needing to focus on changing beliefs rather than merely influencing beliefs.

However, one must be careful not to stop at that level of understanding. There is indeed a very sophisticated art and science of advertising with its roots in what is referred to as "persuasion research." "Persuasive methods" and "message manipulation" have been subject to rigorous research and much is known about the preferred ways of boosting tobacco sales through influencing and changing consumer beliefs. Those of us in health research are generally unaware of this body of knowledge and how it has been used to create and sustain smokers. We become familiar with such research so we can use it in our arsenal of tools aimed at counter-advertising tobacco. As DiFranza and Pollay write, we need to review "Cigarette Package Design: [as] Opportunities for Disease Prevention." Recently Canada seized on this opportunity and currently uses cigarette packaging to communicate health messages; a similar approach is proposed for Australia. Rather than taking the usual advertising approach of getting the consumer to believe that the product has desirable attributes, and avoid having the consumer believe that the product has seriously undesirable attributes, this counter-advertising approach presents the undesirable attributes clearly and convincingly. The recent Canadian advertising of the health consequences of tobacco is particularly graphic and reportedly effective as well. For example, pictures of diseased lungs are included prominently on the packaging. Also, expressions such as "cigarettes can make you ...," together with a cartoon depicting a limp cigarette, are used to get the attention of virile adolescents and young adults who do not realize the risk of impotence. This is an effective approach that governments world-wide should consider to counter-advertise.

While one may argue that the "warning labels" found on tobacco products in the United States and other countries have also taken the approach of presenting the undesirable product characteristics, it has not been the most prominent of messages; indeed, "warning labels" generally appear in smaller print and are not prominently displayed. As a result some consumers may not be drawn to read the warning. The Canadian approach, as well as the approach anticipated in Australia, is a model that should be replicated in other countries. Such an approach may require legislation; thus lobbying needs to be done in coordination with government and private health agencies and organizations, government officials, and elected representatives.

Tobacco advertisers have been greatly influenced by research indicating that "the influence of advertising on receivers' attitudes toward a given brand or product comes about not only through receivers' beliefs about the product's characteristics, but through the receivers' evaluation of the product itself (the receivers' "attitude-toward-the-ad"). As receivers have more favorable evaluations of the advertising, they come to have more favorable evaluations of the product being advertised. And this effect occurs over and above the advertising's effects on product beliefs."* We have seen this phenomenon on a large scale each year in the United States during the "Super-Bowl." Advertisers spend millions of dollars for a spot during the television broadcast, not only to tout their product, but also to entertain the public with the advertisement itself. Placing a product in an entertainment venue becomes a powerful element for product promotion.

Since tobacco products are prohibited from television advertising in the United States, they do not appear in the Super Bowl advertising spots. However, the lesson on the power of advertising is still important. This should move public health advocates to use not only the strength of counter-advertising, but also an appealing advertising campaign that will evoke favorable evaluations of the advertising ("attitude-toward-the ad") by the viewing public. Public health must begin to fight back with the same psychological methods that have proven successful for tobacco advertising. These are powerful and effective methods and must be redirected to promote the health of the public.

We may conclude that advertising is not only an art, but also a science with a strong empirical base that can persuade consumers to influence or change beliefs by advertising attention to the perceived desirable attributes of the product as well as offering an advertising approach that is of itself appealing and attractive. As such tobacco advertising is not unlike the maiden in the verse from Longfellow found in the beginning of this article. "Beware! Beware! Trust her not, She is fooling thee."

Daniel R. Longo, Sc.D.

University of Missouri-Columbia

MA 306 Medical Sciences Building

Columbia, Missouri 65212 USA

## Competing interests

The authors declare that they have no competing interests.

## Note

*O'Keefe DJ. Persuasion: Theory and Research. London: Sage Publications, 1990, p. 53–54.

